# Immunofluorescence Evaluation of Myf5 and MyoD in Masseter Muscle of Unilateral Posterior Crossbite Patients

**DOI:** 10.3390/jfmk5040080

**Published:** 2020-11-07

**Authors:** Giovanna Vermiglio, Antonio Centofanti, Guglielmo Ramieri, Michele Tepedino, Michele Runci Anastasi, Antonio Girolamo Micali, Alba Arco, Maria Grazia Piancino

**Affiliations:** 1 Department of Biomedical and Dental Science and Morphofunctional Imaging, University of Messina, 98166 Messina, Italy; gvermiglio1@unime.it (G.V.); amicali@unime.it (A.G.M.); aarco@unime.it (A.A.); 2 Department of Surgical Science, University of Turin, 10124 Torino, Italy; guglielmo.ramieri@unito.it (G.R.); mariagrazia.piancino@unito.it (M.G.P.); 3 Department of Biotechnological and Applied Clinical Science, University of L’Aquila, 67100 L’Aquila, Italy; m.tepedino@hotmail.it; 4 I.R.C.C.S. (Istituto di Ricovero e Cura a Carattere Scientifico), Centro Neurolesi ‘Bonino Pulejo’, 98100 Messina, Italy; miki_runci@hotmail.it

**Keywords:** unilateral posterior crossbite, masseter muscle, Myf5, MyoD, PAX7, immunofluorescence

## Abstract

A unilateral posterior crossbite is a malocclusion where the low activity of the affected masseter muscle is compensated by the contralateral muscle hypertrophy. It is still unknown if, in the same condition, myogenesis with new fibre formation takes place. Aim: the aim of the present study was to evaluate the expression of myogenesis markers, such as Myf5 and MyoD, in masseter muscles of unilateral posterior crossbite patients. Materials and methods: biopsies from fifteen surgical patients with unilateral posterior crossbites have been analysed by immunofluorescence reactions. The results show the expression of Myf5 and MyoD in the contralateral muscle but not in the ipsilateral one. Moreover, statistical analysis shows the higher number of satellite cells in the contralateral side if compared to the ipsilateral one. Conclusions: these results suggest that in contralateral muscle, hyperplastic events take place, as well as hypertrophy.

## 1. Introduction

A unilateral posterior crossbite is a type of malocclusion determined by an abnormal relationship between the upper and lower dental cusps of the molar and premolar regions, and by a mandibular shift towards the crossbite side [1,2].

Subjects affected by unilateral posterior crossbites present altered chewing cycles and altered masticatory muscle functions [1,3,4]. Moreover, electrophysiological studies have shown that the muscle on the affected side, named “ipsilateral”, works less than the contralateral muscle, and the contralateral muscle seems to work at a high level, probably to compensate the altered contractile function of ipsilateral muscle.

Little morphological data exist on masticatory muscles during crossbite disease, and most of them have been performed on animal models [5,6,7]. Our morphological investigations, performed on humans, have demonstrated—in contralateral muscles—an increased thickness of muscle fibres and an increase in integrins and sarcoglycan expression [8,9]. Integrins are proteins that guarantee a correct muscular development during somitogenesis [10,11], and sarcoglycans are transmembrane glycoproteins that play a key role in sarcolemma stabilization during muscle contraction [12,13,14,15,16]. Conversely, in the ipsilateral muscle, we have found a strong reduction in sarcoglycans and integrin expression, and a marked fibre alteration in terms of morphology and thickness. All of these data have been explained as the results of hypertrophic response of contralateral muscles due to the high workload, to which it is subjected during unilateral crossbite, and of atrophic or dystrophic response of the ipsilateral muscle due to its reduced contractile activity. It has been supported by data showing deficits in myonuclei and satellite cell numbers in the ipsilateral muscle if compared to the contralateral one, characterized by a high number of myonuclei and a high number of satellite cells.

Although satellite cells have been already examined during unilateral crossbite, no data exists about the expression of myogenesis markers in this pathological condition. The study of myogenesis markers could give more important information about morphological changes taking place in the muscle during malocclusion; in particular, they could support the existence of hyperplastic events with formation of new muscle fibres since myogenesis has been already demonstrated in adult muscles [17,18]. That could be very interesting since the etiopathology of malocclusion is still unclear, and it is not yet known whether the skeletal or muscular defects arise first. On this basis, the aim of the present study was to evaluate the expression of two myogenesis regulators, Myf5 and MyoD, in human masseter muscles during unilateral posterior crossbite disease.

## 2. Materials and Methods

### 2.1. Patients and Ethics

Fifteen surgical patients, 7 men and 8 women, age 30.5 ± 5.5 years (mean ± standard deviation), with unilateral posterior crossbite, 10 on the right side and 5 on the left side, were recruited for the study, and all of them gave informed consent.

The inclusion criteria for the crossbite patient group were: (i) severe class III malocclusion with right or left posterior crossbite of two or more posterior teeth; (ii) complete permanent dentition; (iii) no erupting teeth; (iv) no caries; and (v) no temporomandibular disorders. The exclusion criteria were: no history of connective tissue disorders, myopathies, endocrine disorders, autoimmune disease, bone disease, bleeding disorders.

The work has been conducted in accordance with the Declaration of Helsinki (1964) and it has been performed with the understanding and the consent of the human subjects. The work was also approved by local ethics committee, the Institutional Review Board of the University Hospital Health and Science Complex Turin-Italy.

### 2.2. Muscle Biopsies

Biopsies were obtained under general anaesthesia from the superficial portion of both masseter muscles of patients undergoing orthognathic surgery to reposition one or both jaws in conjunction with orthodontic treatment following the protocol suggested by Boyd et al. [19]. All biopsies were obtained by the same surgeon via an intraoral incision through the mucosa and buccinator muscle, approximately 3 × 3 × 3 mm [20].

### 2.3. Immunofluorescence

The biopsies were fixed in 3% paraformaldehyde in 0.2 M phosphate buffer, pH 7.4, for 2 h at room temperature. They were then washed extensively with 0.2 M phosphate buffer, pH 7.4, and then with phosphate-buffered saline (PBS), containing 12 and 18% sucrose. The samples were snap-frozen in liquid nitrogen and 20-μm sections were prepared in a cryostat for use in a protocol to perform immunofluorescence [21,22,23,24,25]. The sections were placed on glass slides that were coated with 0.5% gelatin and 0.005% chromium potassium sulphate.

To block non-specific binding sites and to permeabilize the membranes, the sections were pre-incubated with 1% bovine serum albumin (BSA), 0.3% Triton X-100 in PBS at room temperature for 15 min. Finally, the sections were incubated with primary antibodies. The following primary antibodies were used: anti-PAX7, diluted 1:100, anti-Myf5, and anti-MyoD. PAX7 and MyoD were demonstrated using Texas Red-conjugated IgG (Jackson ImmunoResearch Laboratories, Inc., West Grove, PA, USA); Myf5 was detected using Fluorescein isothiocynate (FITC)-conjugated IgG (Jackson ImmunoResearch Laboratories, Inc., West Grove, PA, USA).

After numerous rinses in phosphate buffer, PBS sections were incubated with DAPI and diluted 1:1000 in PBS (Sigma Aldrich, St. Louis, USA) for 10 min at room temperature to label nuclei. Slides were finally washed in PBS and sealed with mounting medium. The sections were then analysed and images acquired using a Zeiss Confocal Laser Scanning Microscope 5 (CLSM5, Carl Zeiss, Jena, Germany) confocal laser scanning microscope. All images were digitalized at a resolution of 8 bits into an array of 2048 × 2048 pixels. Optical sections of fluorescent specimens were obtained using a helium-neon (HeNe) laser (wavelength, 543 nm) at a 62 s scanning speed, with up to eight averages; 1.50 μm sections were obtained using a pinhole of 250. For each reaction, at least 100 individual fibres were examined. Contrast and brightness were established by examining the most brightly labelled pixel sand, choosing the settings that allowed clear visualization of the structural details, while keeping the pixel intensity at its highest (∼200). Each image was acquired within 62 s, in order to minimize photodegradation.

### 2.4. Statistical Analysis

We analysed random confocal microscope pictures obtained from all patients. In detail, we counted the PAX7 positive cells present in 150 fibres for ipsilateral and 150 fibres for contralateral muscle; we identified these cells by DAPI coloration and PAX7 red fluorescence pattern. After that, we calculated the mean of nuclei for fibres, both for ipsilateral and contralateral muscles, and we compared the mean by Student’s t-test. The means for fibres are shown in graphics.

## 3. Results

### 3.1. Immunofluorescence and Contralateral Muscle

**Figure 1:**
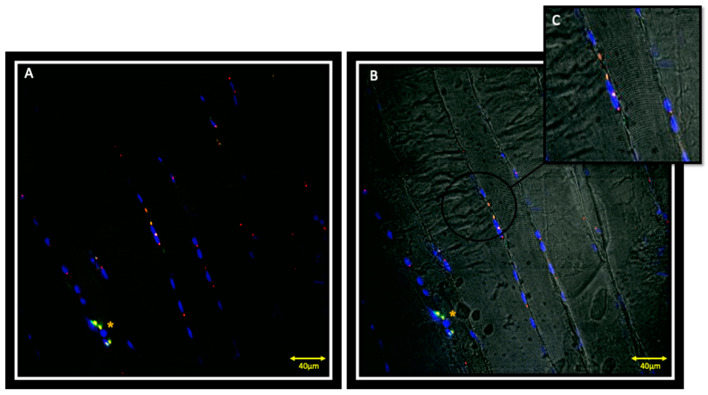
Images of immunofluorescence reactions performed on contralateral masseter muscle; it is possible to observe satellite cells that co-express both PAX7 (red channel) and Myf5 (green channel) (**A**,**B**). (**C**) sub-figure is an high magnification of PAX7 and Myf-5 positive cells. The yellow asterisk indicates a small group of possibly proliferating PAX7 and Myf-5 positive cells. The yellow fluorescence is obtained from the merge of red and green channels. Nuclei are stained with DAPI (blue channel).

**Figure 2:**
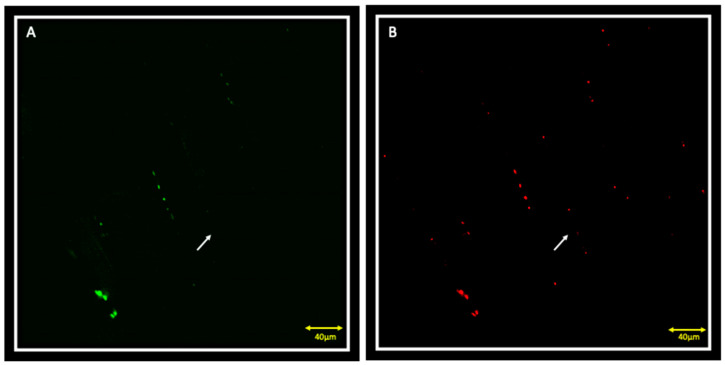
Splitting of channels from Figure 1 with green channel corresponding to Myf5 (**A**) and red channel corresponding to PAX7 (**B**). Results show the presence of cells that are positive for PAX7 (**B**, white arrow) but negative for Myf5 (**A**, white arrow).

Double localization reaction between PAX7 and Myf5 in contralateral muscle showed the presence of PAX7 positive cells that also express Myf5 (Figure 1A,B); these cells, located between the sarcolemma and basal lamina, are satellite cells that have been activated to proliferate (Figure 1C). It is also possible to observe PAX7 and Myf5 positive cells that seem to lose the orderly localization typical of satellite cells between sarcolemma and basal lamina, but are organized in small groups of, possibly, proliferating cells (Figure 1, asterisks). Moreover, in this small group of cells, the fluorescence pattern of Myf5 appears to be more intense if compared to other Myf5 positive cells, suggesting that the intensity of expression is greater in the proliferating cells than those that have only been activated. It is also possible to observe cells that are positive for PAX7 but negative for Myf5 (Figure 2A,B, white arrows), corresponding to quiescent myoblasts.

**Figure 3:**
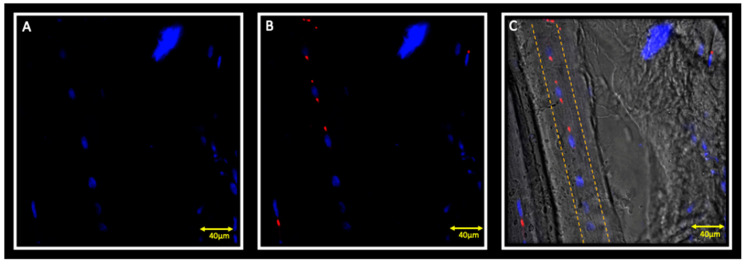
Immunofluorescence reaction in double localization performed on the contralateral masseter muscle. Pictures show muscle fibre characterized by cells that are positive for MyoD (**B**) but negative for Myf5 (**A**); these cells start fusing to form young myotubes, characterised by centrally located nuclei (**C**, dashed line). Nuclei are stained with DAPI (blue channel).

Double localization reaction between MyoD and Myf5 show cells that are positive for MyoD but negative for Myf5 (Figure 3A–C); these cells start fusing to form young myotubes, characterised by centrally located nuclei (Figure 3C, dashed line); moreover, in these fibres, we can observe the absence of any type of cell in the periphery of fibres, as evidenced by the absence of nuclei that are marked with DAPI (blue channel).

### 3.2. Immunofluorescence and Ipsilateral Muscle

**Figure 4:**
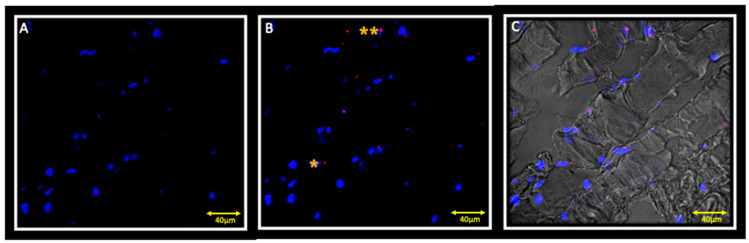
Immunofluorescence reaction in double localization performed on ipsilateral masseter muscle. Results show no Myf5 positive cells (**A**) and a small number of PAX7 (red channel) positive cells (**B**); double yellow asterisks indicate PAX7 positive cells. Figure (**C**) shows the transmitted light.

**Figure 5:**
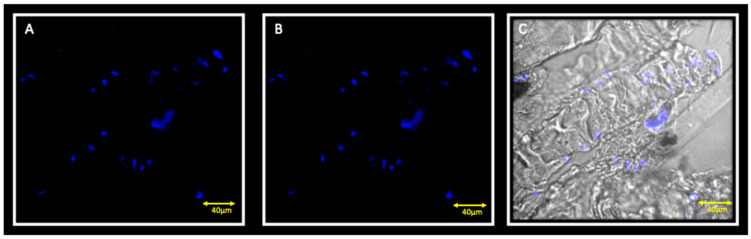
Immunofluorescence reaction in double localization performed on the ipsilateral masseter muscle showing cells that are negative both for Myf5 (**A**) and MyoD (**B**). Nuclei are stained with DAPI (blue channel). The (**C**) pictures show the transmitted light.

Double localization reaction between PAX7 and Myf5 show the presence of few cells that are positive for PAX7, but negative for Myf5 (Figure 4A,B). Double localization reaction between Myf5 and MyoD show cells that are negative for both Myf5 and MyoD (Figure 5 A,B).

### 3.3. Statistical Analysis

**Figure 6:**
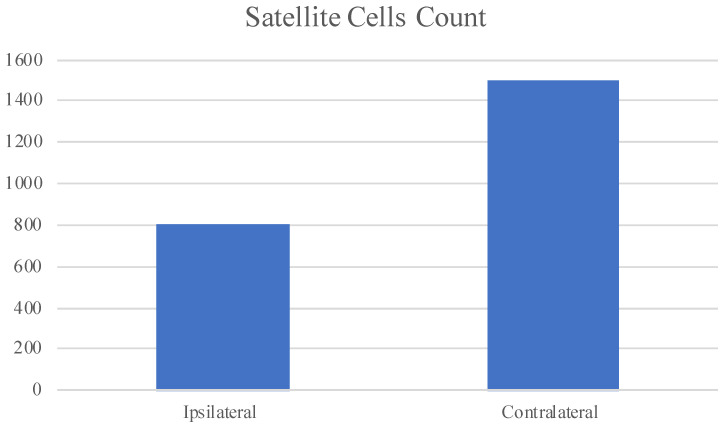
Graphic based on PAX7 positive cells count in 150 fibres, both for ipsilateral and contralateral muscles. Results show a lower number of satellite cells in the ipsilateral muscle if compared to the contralateral one.

Statistical analysis results showed a greater number of PAX7 positive cells in the contralateral muscle if compared to the ipsilateral; the difference between the means is statistically significative with a *p*-value < 0.05. We show statistical analysis results by graphic (Figure 6).

## 4. Discussion

The unilateral posterior crossbite is an asymmetric malocclusion characterized by alterations of morpho-functional characteristics of masticatory muscles [26]. Piancino et al. [1] have shown by electromyography that when chewing on the crossbite side, the ipsilateral masseter presents a reduced activity, while on the contralateral side, the masseter contractile activity is unaltered or increased. Our previous morphological investigations have shown in contralateral masseter an increase in muscle proteins, as sarcoglycans and integrins, and an increase in number of myonuclei and satellite cells, if compared to the ipsilateral one [9]. Satellite cells are muscle stem cells interposed between the sarcolemma and the basement membrane of the muscle fibres; these are cells in a quiescent state [27], but they are induced to proliferate by injury or by increased muscle activity [28]. The activated satellite cells are involved in myonuclei donation to pre-existing fibres, determining an increase in fibre size and in muscle proteins synthesis. On this basis, we have hypothesized that the satellite cells, activated by the high workload, donate nuclei to pre-existing fibres determining the hypertrophy of contralateral muscle. Although, we do not know if even myogenesis with new fibre formation is induced.

In the present study, we examined the expression pattern of two myogenic regulatory factors—Myf5 and MyoD—in order to verify if myogenesis take place.

Our results obtained from contralateral muscle showed the presence of PAX7 positive cells that also express Myf5; these cells, located between the sarcolemma and basal lamina, are satellite cells that have been activated to proliferate. We have also observed PAX7 and Myf5 positive cells that seem to lose the orderly localization typical of satellite cells between sarcolemma and basal lamina, but are organized in small groups of, possibly, proliferating cells that express more Myf5 if compared to other Myf5 positive cells, suggesting that the intensity of expression is greater in the proliferating cells than those that have only been activated. Cells that were positive for PAX7 but negative for Myf5 have also been observed, and they correspond to quiescent myoblasts. These data are in accordance with studies that have demonstrated that, after activation, and in the early phases of proliferation, satellite cells express Myf5 [29]. Successively, cells downregulate Myf5 expression and express MyoD necessary to the fusion of satellite cells to form new muscle fibres [30,31,32]. That is supported by the present results, showing cells that are positive for MyoD but negative for Myf5; these cells are shown to be centrally located as in the primary myotube and no nuclei in fibre periphery are detectable. That strongly supports how we are observing newly formed fibres and not pre-existent fibres.

In the ipsilateral muscle, we found only PAX7 positive cells, but all negative, both for Myf5 and MyoD. Since it has been demonstrated that quiescent satellite cells do not express Myf5 and MyoD, we can speculate that, in the ipsilateral muscle, no myogenesis take place. That is in accordance with our previous data and suggests that the strongly reduced contractile activity no longer represent an adequate stimulus for the satellite cells activation, proliferation and expression of myogenesis regulators. We also performed a statistical analysis that showed a greater number of PAX7 positive cells in the contralateral muscle if compared to the ipsilateral, supporting what we have already shown; the difference between the means is statistical significative.

## 5. Conclusions

All of these data provide interesting information about the morphofunctional changes that occur in the muscle during crossbite. The existence of hyperplastic events, other than hypertrophic events, emphasized the impact that malocclusion can have on the musculoskeletal system. It also highlights the attention that must be paid, especially in younger patients, to the identification of a targeted therapy that could prevent worsening, derived from the hyperactivation of a hypertrophic muscle on one side and the inactivity of an atrophic or dystrophic muscle on the other side.
